# Cross-Cultural Adaptation, Validity, and Reproducibility of the Mediterranean Islands Study Food Frequency Questionnaire in the Elderly Population Living in the Spanish Mediterranean

**DOI:** 10.3390/nu10091206

**Published:** 2018-09-01

**Authors:** Ana Zaragoza-Martí, Rosario Ferrer-Cascales, José Antonio Hurtado-Sánchez, Ana Laguna-Pérez, María José Cabañero-Martínez

**Affiliations:** 1 Department of Nursing, Faculty of Health Science, University of Alicante, 03690 Alicante, Spain; ana.zaragoza@ua.es (A.Z.-M.); ja.hurtado@ua.es (J.A.H.-S.); ana.laguna@ua.es (A.L.-P.); mariajose.cabanero@ua.es (M.J.C.-M.); 2 Department of Health Psychology, Faculty of Health Science, University of Alicante, 03690 Alicante, Spain

**Keywords:** validity, reproducibility, FFQ, elderly, nutrition

## Abstract

The objective of this study was to perform cross-cultural adaptation of the Mediterranean Islands Study Food Frequency Questionnaire (MEDIS-FFQ) and to evaluate its reproducibility and validity in a population over 60 years of age in the Spanish Mediterranean. Three hundred forty-one people completed the food frequency questionnaire (FFQ), which was administered twice (FFQ1 and FFQ2) with nine 24-h dietary recalls (24-HDRs) over a nine-month period to assess its reproducibility and validity. Cross-cultural translation and adaptation were performed according to the International Society for Pharmacoeconomics and Outcomes Research (ISPOR) guidelines and included direct translation, back-translation, and a pilot comprehension test. Reproducibility was evaluated with Pearson’s and interclass correlation coefficients. Validity was estimated using correlations between the FFQ food groups and the 24-HDR mean. The levels of agreement and misclassification were expressed as the proportions of individuals classified by comparing the estimated information from the FFQ2 and the 24-HDR. Reproducibility correlation coefficients ranged from *r* = 0.44 to *r* = 0.90. Validity indices ranged from 0.71 to 0.99. More than 80% of the subjects were classified in the same quartile on both instruments. The kappa statistic showed a moderate to high level of agreement (0.70–0.95) between the two instruments. In conclusion, the MEDIS-FFQ showed good reproducibility and validity in estimating the nutrient intake of the elderly population in the Spanish Mediterranean.

## 1. Introduction

The dietary habits of the elderly population directly influence disease development and progression as well as prevention and treatment [1,2]. Epidemiological studies have shown that a Mediterranean dietary pattern with a high intake of fruits and vegetables, legumes, whole grains, and olive oil, moderate intake of dairy products, low intake of meat and meat products, and moderate intake of alcohol with meals are directly related to reduction of overall mortality and cardiovascular mortality in particular [3]. In addition, the Mediterranean diet is associated with a lower risk of suffering chronic degenerative diseases, such as obesity, cardiovascular diseases, some types of cancer, and cognitive decline [4,5,6].

For this reason, quantifying food intake in this population group is vitally important for clarifying their dietary habits and nutrient intake and the relationships of these factors with health markers [1,7]. In this sense, food frequency questionnaires (FFQs) are essential tools in epidemiological studies to determine the relationship between diet and diseases over a long period of time [8,9]. Using this tool, the frequency of food group intake and typical serving sizes can be determined over a period of time. This information allows quantification of both the consumption frequency and the energy, macronutrient, and micronutrient intakes of each individual [10]. In addition, FFQs are easy to use, with relatively low, and can be administered to large population groups [11].

However, FFQs are very sensitive to different lifestyles, eating habits, and dietary preferences of the study population [2,12]. The use of inappropriate foods in a FFQ list may result in an underestimation of nutrients due to the omission of important items. Therefore, the reproducibility and validity of a FFQ must be evaluated in the population under study [5,6,13].

In Spain, numerous studies have investigated the validation and reproducibility of FFQs in different population groups, including pregnant women, children, and the elderly [14,15,16]. However, few studies have focused on validation of an FFQ adapted to the elderly Mediterranean population that includes foods from the Mediterranean diet, such as different oils, garlic, onion, nuts, and different types of fish [16]. In particular, a validation study of an FFQ in the Prevention with Mediterranean Diet (PREDIMED) study was conducted in the elderly population [16]. This FFQ consists of 137 items, 19 items more than in the original questionnaire [17]. In this regard, age-related factors that may affect the ability to obtain valid information about food intake should be considered, such as hearing, vision, and memory loss. Interview length is essential because older people may need more time to respond and feel more fatigued or frustrated when responding, and these factors can contribute to a lower response rate [7]. Therefore, the development of a simpler and shorter FFQ adapted to the needs of the elderly population is appropriate for the aim of reducing possible biases.

Therefore, the objective of this study is to perform a cross-cultural adaptation of the Mediterranean Islands Study Food Frequency Questionnaire (MEDIS-FFQ) and to evaluate its reproducibility and validity in a population over 60 years of age living in the Spanish Mediterranean.

## 2. Experimental Methods

### 2.1. Study Population

This study included 341 people, all of whom were over 60 years of age and were living in the Spanish Mediterranean area of Alicante. Four hundred twenty-six people were invited to participate in the study; a response rate of 80% resulted in a final sample of 341 participants. All participants were volunteers and signed an informed consent form prior to participation in the study. The participants were selected from an environment close to the interviewers’ location using a snowball strategy. The study excluded all subjects who were dependent in activities essential for daily life, with three or more errors in the Pfeiffer test (Short Portable Mental Status Questionnaire), and who could not read or write.

### 2.2. Study Design

**Figure 1:**
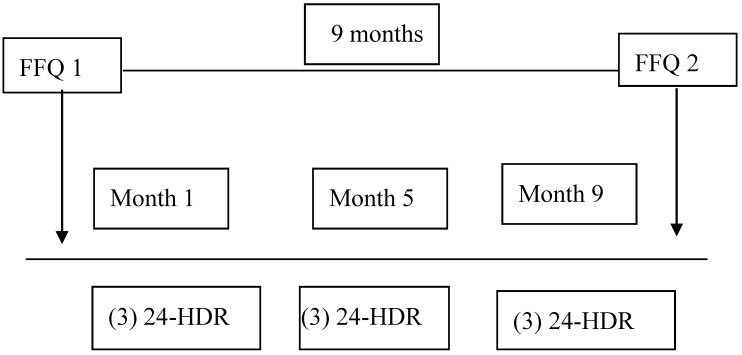
Study design. 24-HDR: 24-h dietary recall; FFQ: food frequency questionnaire.

The study was conducted over a nine-month period. At the beginning and at the end of this period, the participants completed a FFQ (FFQ1 and FFQ2). Over the nine months, food intake for 9 days was estimated with 24-h dietary recalls (24-HDRs) during three time-spaced periods to control for seasonal variation in food consumption. During each period, food consumption was estimated for three consecutive days, including two working days and a holiday. Figure 1 shows the study design.

### 2.3. Cross-Cultural Translation and Adaptation

The cross-cultural translation and adaptation were performed according to the International Society for Pharmacoeconomics and Outcomes Research (ISPOR) guidelines [18] and included direct translation, back-translation, and a pilot comprehension test.

#### 2.3.1. Direct Translation

After receiving authorisation from the original authors to adapt the original version into Spanish, the original questionnaire was translated into Spanish by two independent bilingual translators. Each translator worked separately following specific instructions and was asked to indicate the level of difficulty of the translation (1 = no difficulty to 10 = highest difficulty). They were also asked to evaluate the conceptual equivalence and indicate the type of changes introduced as follows: type A (no changes were necessary, and the sentence structure was maintained), type B (the translation was modified to ensure semantic and conceptual equivalence), and type C (some items were not applicable to the cultural context of the destination).

Translations should be semantic and not literal and should emphasise conceptual equivalence without varying the meaning of each item in the original version. The first translation process resulted in two versions in Spanish. These versions were evaluated by members of the research team (MJCM, RFC, AZM), who performed a qualitative assessment of the linguistic, semantic, and cultural equivalence. Finally, an agreement was reached for version 1 by incorporating some modifications for different items to achieve greater conceptual clarity. During the process, the translators noted that the original questionnaire used terms from both American English and British English; this fact was taken into account, and the questionnaire was translated only into British English.

#### 2.3.2. Back-Translation

With this new version, two other bilingual translators were asked to perform the back-translation to obtain two new versions in English. They were asked to assess quantitatively on a scale of 1 to 5 the syntactic and semantic equivalence of the original questionnaire and the back-translation. They were also asked to evaluate conceptual equivalence following the same procedure as the direct translation.

#### 2.3.3. Pilot Comprehension Test

**Figure 2:**
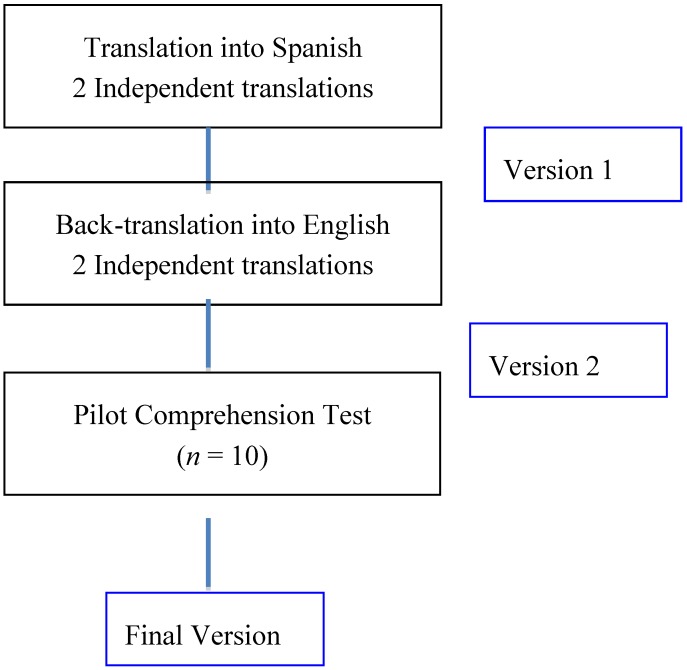
Cross-cultural adaptation of the Mediterranean Islands Study Food Frequency Questionnaire (MEDIS-FFQ).

Once the first draft was prepared, an initial test was performed on a random sample (*n* = 10) to check comprehension, acceptability, language use, and feasibility. The questionnaire was administered in a personal interview by dieticians/nutritionists trained in questionnaire administration. Once the questionnaire was completed by the participants, the objections, modifications, and suggestions considered adequate were recorded and agreed upon by the research team. A final version was obtained by consensus among all researchers. Figure 2 shows the outline of the cross-cultural translation and adaptation process.

### 2.4. Instruments and Diet Evaluation

#### 2.4.1. MEDIS-FFQ

**Figure 3:**
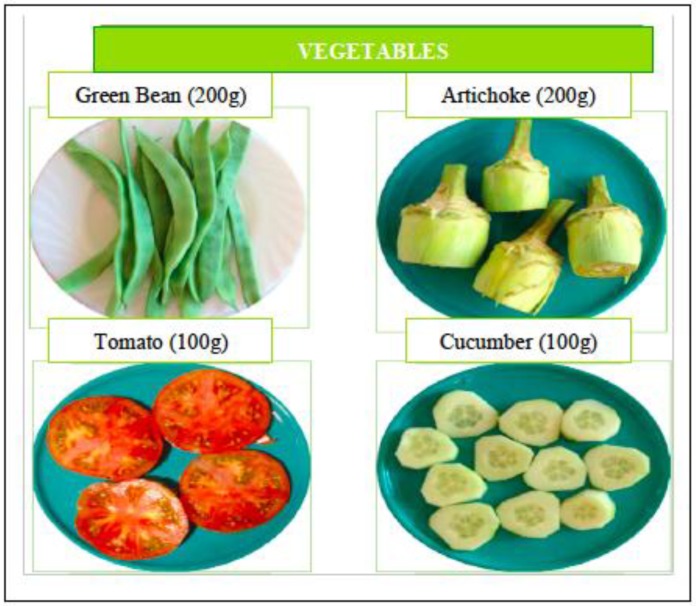
Example image of the photo album.

The MEDIS-FFQ was developed and validated to evaluate the dietary habits of elderly people living in Mediterranean areas in the “the MEDIS (Mediterranean Islands Elderly) study” [19]. The MEDIS study was a longitudinal study with 5- and 10-year follow-up of health and nutrition in people over 65 years of age living in the Mediterranean islands. The MEDIS-FFQ has been validated for the elderly population living in the Mediterranean islands (Cyprus, Peloponnese, Greece, Attica, and Thrace). This questionnaire includes several food and drink groups (11 groups) that are normally consumed in Mediterranean countries (dairy products, cereals and starchy foods, meat and meat products, fish, legumes and traditional dishes, vegetables, fruits and nuts, snacks, sweets, drinks, and fats). The questionnaire also evaluates serving sizes (small, medium, or large) and specifies the type of bread (whole wheat or white), fat (olive oil, margarine...), cheese, or drink consumed. The consumption frequency refers to the previous year, and the frequencies reflected are daily consumption (once per day or more than twice per day), weekly consumption (one to two times per week and three to six times per week), monthly consumption (from one to three times per month), and no consumption or occasional consumption [20]. To quantify seasonal food consumption (fruits and vegetables), the participants were asked to detail the consumption frequency of those foods during the season. To help the participants quantify their actual food intake, a photo album was developed for all items of the FFQ with actual serving sizes (Figure 3).

#### 2.4.2. 24-h Dietary Recall

All participants interviewed completed three 24-HDR assessment two days during the week (Monday to Friday) and one day on a weekend (Saturday or Sunday). This process was performed in triplicate every four months. During the interview, each participant described in detail the type and the amount of food and drink ingested during the previous 24 hours. The subjects were not informed about the study until the afternoon before the interview. All combined dishes became a single meal, and the weight of each food was calculated according to the ingredients and serving sizes. To ensure that those foods recorded by the 24-HDR were comparable with the foods recorded by the FFQ, each food recorded in the 24-HDR was assigned to the different food groups defined by the FFQ. All records were checked individually with the participants to resolve ambiguities. The interviewer was the same for each participant throughout the study period.

### 2.5. Procedure

Variables were measured by trained personnel with experience in administering questionnaires. All questionnaires were completed in a systemised manner by the interviewers at the time of the interviews using an ad hoc booklet. Prior to the start of the study, a pilot study was conducted in a small sample with the aim of verifying the viability and administration procedure. The participants were interviewed on three occasions between the months of December and July. In the first interview, they were given the FFQ1 and a three-day 24-HDR. In the second interview, the second three-day 24-HDR was administered. Finally, in the third interview, they were given the FFQ2 and another three-day 24-HDR.

### 2.6. Ethical Considerations

The present study was approved by the Ethics Committee of the University of Alicante (UA-2016-02-11). This study was conducted according to the criteria of the Declaration of Helsinki and the Good Clinical Practice Guidelines of the European Union. To protect the strict confidentiality of the data, anonymous codes were assigned to identify the study participants. Once the information was collected, a member of the research team entered the data into the study database. Any personal information of the participants that could be used for identification was not included in the database. All study participants read and signed an informed consent form prior to participation in the study.

### 2.7. Calculation of Food Quantities and Nutrient Estimates

The responses obtained from the FFQ on the consumption frequency of each food were unified into daily frequencies using the mean value of each category. The coefficients 0.0, 0.07 (2/30), 0.21 (1.5/7), 0.64 (4.5/7), 1.0, and 2.5 were used to indicate the frequencies “never or almost never”, “1–3 times per month”, “1–2 times per week”, “3–6 times per week”, “once per day”, and “two or more times per day”, respectively. Next, these coefficients were multiplied by each food item quantity in grams to obtain the amount consumed daily in grams [21]. The individual estimates of daily food intake and nutrients were calculated using food composition tables validated for the Spanish population and adjusted for the edible portion [22].

### 2.8. Statistical Analysis

Descriptive analyses were used to describe the characteristics of the participants and their mean food, energy, and nutrient intakes. All food and nutrient intake variables followed a normal distribution, and thus, parametric tests were used in this case. Significant differences in the intake, nutrients, and food groups between the FFQ1 and FFQ2 and between the FFQ and 24-HDR were determined with the Wilcoxon test.

#### 2.8.1. Reproducibility

Reproducibility (test-retest) was evaluated with the correlation of the mean intake from the FFQ1 and FFQ2 using Pearson’s correlation coefficients. Interclass correlation coefficients were calculated by comparing energy and nutrients between the two FFQs. Correlation coefficients > 0.4 indicate acceptable agreement [7].

#### 2.8.2. Validity

Validity was evaluated by comparing the mean daily intake of gross nutrients and that adjusted for the daily caloric intake between the FFQ and the mean of the nine 24-HDRs. Subsequently, the correlations between food groups of the FFQ and the 24-HDR mean were estimated. These correlations were calculated using Pearson’s correlation coefficients according to their distributions. The levels of agreement and misclassification were expressed as the proportions of individuals classified in the same quartile, an adjacent quartile, and the extreme quartile between nutrient intake estimated by the FFQ2 and the 24-HDR mean. The kappa statistic (*K*_w_) was calculated by comparing intake quartiles for each nutrient in the FFQ and the 24-HDR. The following values were used to evaluate the level of agreement between dietary recording methods: ≥ 0.80 indicates very good agreement, *K*_w_ = 0.61–0.80 indicates good agreement, *K*_w_ = 0.41–0.60 indicates moderate agreement, *K*_w_ = 0.21–0.40 indicates poor agreement, and *K*_w_ ≤ 0.20 indicates very poor agreement.

To visualise the agreement between the two methods in terms of absolute intake, we used the Bland–Altman method, which plots differences in intakes between the two methods (FFQ-24-HDR) versus the mean intake of the two methods ((FFQ2 + 24-HDR)/2). The limits of agreement (LOAs) of the Bland–Altman plots were determined according to the mean score (the mean of the difference between the dietary pattern scores) and the (95%) LOA (mean ± 1.96 standard deviation of the differences).

All calculations were performed with the SPSS version 20.0 statistical (IMB Corp, Alicante, Spain) package, and the statistical significance for all tests used was established at 0.05 bilaterally.

## 3. Results

Three hundred forty-one subjects participated in the study, of whom 56% (191) were women. The mean age was 72.59 ± 59 years, 63.94% (218) were married, and the mean of the Body Mass Index (BMI) was 27.61 ± 6.72 kg/m^2^. The prevalence of smokers was 23.75% (81), and that of drinkers was 56.0% (191) (Table 1).

### 3.1. Cross-Cultural Adaptation

During the cross-cultural adaptation process, the translators rated the translations and back-translations of the questionnaire items with a medium-low difficulty level. Items with higher levels of difficulty were item 35 (spinach-rice/cabbage-rice), item 36 (pastitsio/moussaka/papoutsakia), and item 57 (sweets made in a tray). The changes made were type C in 3.75% of cases (*n* = 3), type B in 27.5% (*n* = 22) of cases, and type A in 68.75% (*n* = 55) of cases.

In the comprehension study, the cognitive interviews showed very few comprehension problems. A comprehension problem was found only for three items (item 3 (yellow cheese)), item 12 (zwieback), and item 55 (pies)). These problems were solved by making the syntactic and lexical changes necessary to improve their understanding. Based on the data obtained, the research team reviewed the pilot version of the questionnaire; once the proposed modifications were agreed upon, the process was closed, resulting in the final version of the MEDIS-FFQ in Spanish.

### 3.2. Nutrient and Food Group Intakes

The means and standard deviations of the total energy, nutrient, and food group intakes estimated by the FFQ and the means of the nine 24-HDRs are shown in Table 2 and Table 3. In the food intake estimates, there are no clear patterns of underestimation or overestimation. Conversely, the estimates of nutrient intake by the 24-HDR were higher than those obtained by the FFQ 1 and lower than those obtained by the FFQ 2 except for cholesterol, calcium, vitamin B1, vitamin B2, and vitamin A.

### 3.3. Reproducibility

Table 4 shows the interclass correlation coefficients between the FFQ1 and FFQ2. The correlation coefficients range from 0.30 to 0.91 for potassium and vitamin D, respectively. The energy-adjusted model tends to reduce the correlation coefficients between FFQ1 and FFQ2 except for iodine. Regarding the level of agreement, proteins showed a low correlation (30.7%), whereas lipids, vitamin B6, and vitamin B12 had very high levels of agreement. The remaining nutrients had moderate to high agreement.

### 3.4. Validation

Table 5 shows the intakes estimated by the FFQ2, the mean of the nine 24-HDRs, and the correlation coefficients. Analysis of Pearson’s correlation coefficients for the 24-HDR and FFQ2 results showed the highest correlations for lipids (*r* = 0.99), sodium (*r* = 0.99), vitamin B6 (*r* = 0.99), and vitamin D (*r* = 0.99). The lowest coefficients corresponded to vitamin B1 (*r* = 0.71), vitamin A, and vitamin E (*r* = 0.78). When adjusted for energy (kcal), Pearson’s correlation coefficients remained stable for most nutrients. Among the nutrients, the adjusted correlations increased compared to the unadjusted correlations for polyunsaturated fatty acids (*r* = 0.80), carbohydrates (*r* = 0.89), sodium (*r* = 0.99), vitamin A (*r* = 0.78), vitamin D (*r* = 0.99), and vitamin C (*r* = 0.98). The differences in the mean nutrient intakes range from 0.01 for vitamin B6 to 62.90 for potassium.

Cross-classification analysis showed that >85% of the subjects were classified in the same quartile (Table 6). The proportion of subjects classified in the same quartile ranged from 83.53% for vitamin A to 99.88% for sodium. The proportion of subjects classified in the extreme quartile was less than 3%, with the highest value obtained for vitamin B1 (2.06%).

**Figure 4:**
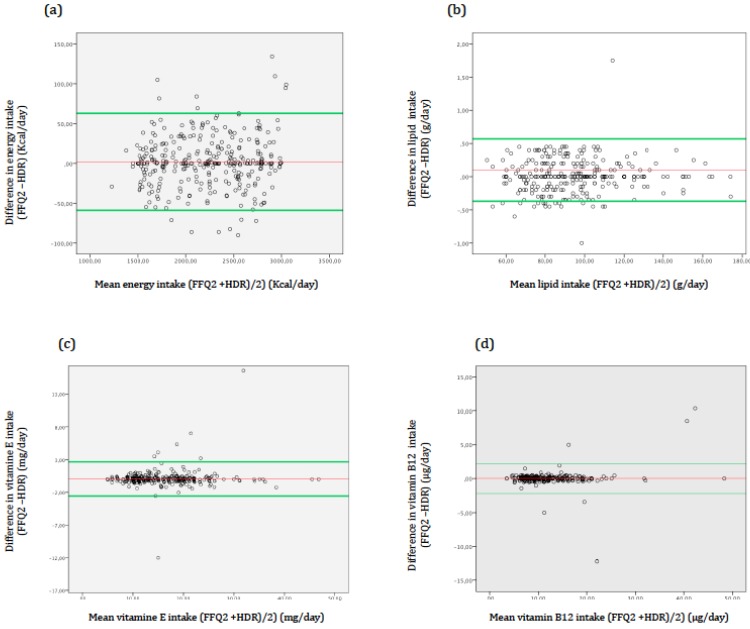
Bland–Altman plots showing the relationship between mean and differences in the daily intake of (**a**) energy, (**b**) lipids, (**c**) vitamin E, and (**d**) vitamin B12 estimated with the second FFQ (FFQ2) and three 24-HDR and the corresponding mean intake estimated by the two methods.

Bland-Altman plots (Figure 4) for energy, total lipids, vitamin E, and vitamin B12 showed a homogeneous dispersion above and below zero in most plots.

## 4. Discussion

### 4.1. Reproducibility

The mean nutrient and food intakes obtained by the FFQ1 and FFQ2 were similar. This finding can be explained by the learning process of the participants throughout the study. The participants were able to improve their food intake estimates beforehand through several personal interviews [11]. The correlation coefficients for reproducibility in our study range from *r* = 0.44–0.90, with the lowest values for vitamin A and iron and the highest for vitamin B12, sodium, and vitamin C. These results are comparable to those found in other studies that have examined the reproducibility of FFQs designed for specific populations, such as those developed for the elderly population (*r* = 0.50–0.82) [16], adult population (*r* = 0.49–0.96) [1], and pregnant women (*r* = 0.2–0.70) [15]. At the international level, the results found in different countries are also in agreement with those of the present study, with correlation coefficients ranging from 0.40 to 0.80 [19,23,24].

Correlation coefficients were also estimated with the energy-adjusted data to control for possible confounding factors. In this case, slight variation was observed with respect to the unadjusted data without significance, as was reported in other studies [17,24,25].

### 4.2. Validation

Our FFQ has good validity. The correlations found after adjusting for energy to control possible confounding effects ranged from 0.71 to 0.99, indicating high validity for accurate determination of food intake. These correlations were lower for vitamin B1 and vitamin A and higher for saturated fats, vitamin B6, and vitamin D. Slightly lower correlations have been found in other studies, with a range from 0.24 to 0.9 [3,16,24,26]. These differences may be caused by the difficulties encountered in estimating serving sizes in other studies. In this sense, our study minimised this bias as much as possible by producing a photo album with the exact serving size included in the FFQ. In addition, life-size food replicas with the actual weight of each serving were used in each interview. Therefore, the correlations found in our study may have been higher.

The results of the validation estimate of the FFQ depend on many factors, including the choice of reference method, the degree of population homogeneity, and the number of recalls. The selection of an appropriate reference method is one of the most important parts of the validation process. Similar to a large proportion of the scientific literature, this study uses 24-HDR (75%) as an appropriate reference method for validation of the FFQ [6,27,28,29,30]. However, the errors of the FFQ and the reference method must be independently measured. Our study estimated validation by comparing the FFQ with the mean of the nine 24-HDRs performed in different seasons of the year, which allowed us to accurately estimate seasonal and weekly variations and obtain a high degree of accuracy in nutrient estimates.

The capacity to classify individuals based on food or nutrient intake is very important for conducting epidemiological studies. The cross-classification of nutrients showed that a high number of participants were in the same quartile (>80%) and a low percentage were in the opposite quartile (<3%). These results demonstrate good agreement between the FFQ and the 24-HDR. Additionally, the kappa index showed a moderate to high level of agreement (0.70–0.95) between the two instruments. Similar results have been found in other studies, with more than 70% of the subjects located in the same or an adjacent quartile [16,26,31,32].

The Bland–Altman method used is the most appropriate method for estimating absolute validity by estimating mean agreement and the limits of agreement. Mean agreement indicates the mean of the difference between the assessment instruments used to determine food intake. This value was approximately equal for the two methods (the FFQ and the 24-HDR), which showed a good degree of absolute validity.

The present study has several limitations. First, the sample was not randomised and included only voluntary participants. Voluntary participants tend to have a better health status and greater motivation, which can apparently increase the reproducibility and validity of the FFQ. Second, due to the lack of a gold standard to measure dietary intake, the 24-HDR was selected as a widely used reference method for validation of FFQs. Good memory is needed for both methods, and therefore, the source of error cannot be the same [21]. To control possible memory bias, life-sized photos of the foods included in the FFQ were shown in each of the interviews. In addition, life-size food replicas with actual weights were provided.

## 5. Conclusions

In conclusion, the MEDIS-FFQ has been shown to be a valid and accurate instrument to estimate nutrient intakes and dietary habits in the elderly population living in the Spanish Mediterranean. Since it is a shorter questionnaire, the quality of the answers is improved because possible limitations due to fatigue or weariness in this age group are diminished. Therefore, this tool is valuable for the performance of epidemiological studies in the elderly population because it allows the identification of different risky eating behaviours and their relationships with health markers.

## Figures and Tables

**Table 1:** Sociodemographic characteristics of the participants in the study of the validity and reproducibility of the MEDIS-FFQ.

	Total (*N* = 341)	Women (*N* = 191)	Men (*N* = 150)
	*N* (%)	*N* (%)	*N* (%)
Age			
Mean	72.59	73.38	71.58
SD	8.29	8.16	8.38
Weight			
Mean (kg)	74.68	70.25	80.32
SD	12.89	11.67	12.18
BMI			
Mean	27.61	27.69	27.52
SD	6.72	4.69	4.79
Years of education			
None	43 (12.60)	30 (15.70)	13 (8.70)
1–5 years	66 (19.51)	38 (19.90)	28 (18.70)
5–10 years	123 (36.12)	68 (35.60)	55 (36.70)
>10 years	109 (32.00)	55 (28.80)	54 (36.00)
Place of Residence			
Urban	271 (79.47)	150 (78.50)	121 (80.70)
Rural	70 (20.53)	41 (21.50)	29 (19.30)
Civil Status			
Married	218 (63.93)	114 (59.70)	104 (69.30)
Widowed	89 (26.10)	60 (31.43)	29 (19.30)
Divorced	12 (3.52)	7 (3.70)	5 (3.30)
Single	10 (2.93)	6 (3.10)	4 (2.70)
Living as a couple	12 (3.52)	4 (2.10)	8 (5.30)
Alcohol Consumption			
No	150 (43.79)	106 (55.50)	44 (29.30)
Yes, usually	32 (9.38)	5 (2.60)	27 (18.00)
Yes, occasional	159 (46.63)	80 (41.90)	79 (52.70)
Tobacco Consumption			
No	257 (75.37)	156 (81.68)	104 (69.30)
Yes, usually	65 (19.06)	30 (15.70)	35 (23.30)
Yes, occasional	16 (4.69)	5 (2.60)	11 (7.30)
Physical Activity			
No	92 (26.98)	56 (29.30)	36 (24.00)
<2.5 h/week	66 (19.35)	42 (22.00)	24 (16.00)
2.5–7 h/week	126 (36.95)	74 (38.70)	52 (34.70)
>7 h/week	57 (16.74)	19 (9.90)	38 (25.30)

SD: Standard Deviation; BMI: Body Mass Index.

**Table 2:** Daily consumption of food groups estimated by the two food frequency questionnaires (FFQ 1 and FFQ 2) and the 24-HDR.

Food Groups	FFQ 1		FFQ 2		24-HDR	
	Mean	SD	Mean	SD	Mean	SD
Dairy products (g)	377.87	233.87	344.08	218.33	356.89	200.33
Starchy foods (g)	213.02	141.67	212.67	77.30	200.89	89.76
Meat (g)	140.04	87.42	140.89	75.28	145.98	77.89
Fish (g)	98.99	75.39	99.71	74.56	97.76	70.54
Legumes (g)	70.52	50.53	69.68	49.87	72.34	45.67
Vegetables (g)	205.45	122.84	207.65	123.45	201.76	121.98
Fruits (g)	475.06	212.4	469.98	201.50	450.98	200.7
Nuts (g)	8.21	4.67	8.09	4.53	7.98	4.56
Sweets (g)	95.52	34.56	90.92	30.12	93.45	30.21
Snacks (g)	30.48	22.24	35.29	25.86	32.12	21.34
EVOO (g)	62.32	38.36	61.97	37.32	60.78	35.45
Alcoholic beverages (g)	98.47	23.34	103.85	24.67	100.34	22.34

EVOO: extra virgin olive oil.

**Table 3:** Daily nutrient intake estimated by the two FFQs (FFQ1 and FFQ2) and the 24-HDR.

Nutrient	FFQ 1		FFQ 2		24-HDR	
	Mean	SD	Mean	SD	Mean	SD
Energy (kcal)	2222.49	455.80	2380.08	1685.59	2301.28	920.15
Proteins (g)	88.52	58.66	87.58	39.29	88.05	37.44
Lipids (g)	96.48	23.03	99.16	54.20	97.82	33.72
Saturated fats (g)	28.97	5.69	29.04	5.78	29.00	5.70
MUFAs (g)	42.08	12.22	40.68	9.99	41.38	10.23
PUFAs (g)	13.84	3.94	13.92	4.07	13.88	3.96
Cholesterol (g)	242.50	69.56	240.13	67.25	241.32	66.63
Carbohydrates (g)	283.99	54.23	284.24	54.72	284.12	54.46
Fibre (g)	31.15	9.35	31.52	10.14	31.33	9.43
Potassium (mg)	3545.14	721.29	3670.94	2518.52	3608.04	1432.14
Sodium (mg)	2677.46	670.22	2687.47	674.38	2682.47	668.83
Calcium (mg)	1198.45	525.36	1175.82	313.76	1187.14	371.66
Iron (mg)	12.16	3.27	12.54	8.41	12.35	5.13
Iodide (μg)	105.69	58.50	106.53	70.30	106.06	51.67
Vitamin B1 (mg)	1.57	1.03	1.53	0.90	1.55	0.73
Vitamin B2 (mg)	3.03	1.58	2.98	1.16	3.00	1.27
Vitamin B6 (mg)	2.51	0.85	2.50	0.85	2.51	0.84
Vitamin B12 (μg)	11.66	5.34	11.70	5.57	11.68	5.34
Vitamin C (mg)	255.63	102.32	255.72	103.77	255.68	101.92
Vitamin A (μg)	1180.73	890.26	1140.96	859.57	1160.84	657.02
Vitamin D (μg)	5.77	2.78	5.78	2.83	5.78	2.79
Vitamin E (mg)	16.09	6.66	16.25	6.60	16.17	6.49

MUFA: monounsaturated fatty acids; PUFA: polyunsaturated fatty acids.

**Table 4:** Reproducibility of the FFQ: Correlation between energy and nutrient intakes estimated by the FFQ1 and FFQ2.

Nutrient	Interclass Correlation Coefficient		Agreement (%) *	Agreement Significance
	Unadjusted	Adjusted †		
Energy (kcal)	0.99	-	55.5	<0.01
Proteins (g)	0.88	0.79	30.7	<0.01
Lipids (g)	0.86	0.85	40.1	<0.01
Saturated fats (g)	0.99	0.81	99.6	<0.01
MUFAs (g)	0.81	0.80	77.8	<0.01
PUFAs (g)	0.97	0.79	89.4	<0.01
Cholesterol (g)	0.89	0.80	42.7	<0.01
Carbohydrates (g)	0.99	0.86	88.7	<0.01
Fibre (g)	0.87	0.78	71.7	<0.01
Potassium (mg)	0.33	0.30	87.6	<0.01
Sodium (mg)	0.99	0.89	40.1	<0.01
Calcium (mg)	0.66	0.64	78.9	<0.01
Iron (mg)	0.45	0.44	56.7	<0.01
Iodide (μg)	0.43	0.45	54.7	<0.01
Vitamin B1 (mg)	0.57	0.56	67.8	<0.01
Vitamin B2 (mg)	0.80	0.81	67.8	<0.01
Vitamin B6 (mg)	0.98	0.87	98.7	<0.01
Vitamin B12 (μg)	0.96	0.90	90.7	<0.01
Vitamin C (mg)	0.96	0.89	78.9	<0.01
Vitamin A (μg)	0.50	0.49	55.6	<0.01
Vitamin D (μg)	0.98	0.91	87.6	<0.01
Vitamin E (mg)	0.98	0.87	89.8	<0.01

* According to Cohen’s kappa statistic. † Adjusted for total energy intake.

**Table 5:** Validation of the FFQ: correlation between energy and nutrient intakes estimated by FFQ2 and the mean of the three 24-HDRs.

Nutrient	Pearson’s Correlation Coefficient		Difference of Means	
	Unadjusted	Adjusted *	Mean	SD
Energy (kcal)	0.97	-	1.97	31.07
Proteins (g)	0.99	0.98	0.26	2.01
Lipids (g)	0.95	0.95	1.34	24.43
Saturated fats (g)	0.99	0.99	0.03	0.54
MUFAs (g)	0.90	0.90	−0.70	4.46
PUFAs (g)	0.78	0.80	0.04	0.61
Cholesterol (g)	0.97	0.97	−1.18	15.52
Carbohydrates (g)	0.87	0.89	0.12	1.58
Fibre (g)	0.97	0.97	0.19	2.50
Potassium (mg)	0.97	0.97	62.90	1174.99
Sodium (mg)	0.89	0.99	5.00	68.27
Calcium (mg)	0.80	0.80	−11.32	221.58
Iron (mg)	0.95	0.95	0.19	3.80
Iodide (μg)	0.84	0.84	0.46	38.89
Vitamin B1 (mg)	0.71	0.71	−0.20	0.64
Vitamin B2 (mg)	0.89	0.89	−0.22	0.56
Vitamin B6 (mg)	0.99	0.99	−0.01	0.11
Vitamin B12 (μg)	0.98	0.98	0.2	1.11
Vitamin C (mg)	0.89	0.98	0.05	15.22
Vitamin A (μg)	0.74	0.78	−19.89	577.95
Vitamin D (μg)	0.88	0.99	0.01	0.32
Vitamin E (mg)	0.76	0.78	0.08	1.36

* Adjusted for total energy intake.

**Table 6:** Cross-classification of quartiles and the kappa index between the FFQ2 and 24-HDRs.

Nutrient	FFQ2 vs. 24-HDR			Kappa	Significance
	Same (%)	Adjacent (%)	Extreme (%)		
Energy (kcal)	95.88	4.12	0.00	0.95	<0.01
Proteins (g)	97.94	2.06	0.00	0.97	<0.01
Lipids (g)	94.12	5.88	0.00	0.92	<0.01
Saturated fats (g)	95.29	4.71	0.00	0.94	<0.01
MUFAs (g)	90.88	7.35	0.59	0.88	<0.01
PUFAs (g)	98.53	1.47	0.59	0.98	<0.01
Cholesterol (g)	96.18	2.94	0.29	0.95	<0.01
Carbohydrates (g)	95.29	2.94	0.59	0.94	<0.01
Fibre (g)	97.65	2.35	0.00	0.96	<0.01
Potassium (mg)	98.53	1.47	0.00	0.98	<0.01
Sodium (mg)	99.88	1.18	0.00	0.97	<0.01
Calcium (mg)	97.65	1.76	0.29	0.96	<0.01
Iron (mg)	96.18	3.53	0.00	0.93	<0.01
Iodide (μg)	97.35	2.05	0.00	0.96	<0.01
Vitamin B1 (mg)	83.53	14.12	2.06	0.78	<0.01
Vitamin B2 (mg)	97.65	4.71	0.00	0.93	<0.01
Vitamin B6 (mg)	89.44	10.00	0.00	0.86	<0.01
Vitamin B12 (μg)	95.00	5.00	0.00	0.93	<0.01
Vitamin C (mg)	95.29	4.41	0.00	0.93	<0.01
Vitamin A (μg)	83.53	15.59	0.59	0.78	<0.01
Vitamin D (μg)	92.35	7.64	0.00	0.89	<0.01
Vitamin E (mg)	87.65	12.03	0.29	0.84	<0.01
